# Development of 30 Novel Polymorphic Expressed Sequence Tags (EST)-Derived Microsatellite Markers for the Miiuy Croaker, *Miichthys miiuy*

**DOI:** 10.3390/ijms12064021

**Published:** 2011-06-15

**Authors:** Tianjun Xu, Dianqiao Sun, Yuena Sun, Rixin Wang

**Affiliations:** Key Laboratory for Marine Living Resources and Molecular Engineering, College of Marine Science, Zhejiang Ocean University, Zhoushan 316000, China; E-Mails: tianjunxu@163.com (T.X.); ssundianqiaod@163.com (D.S.); yuenasun@163.com (Y.S.)

**Keywords:** microsatellite, Expressed sequence tags (ESTs), *Miichthys miiuy*

## Abstract

Expressed sequence tags (ESTs) can be used to identify microsatellite markers. We developed 30 polymorphic microsatellite markers from 5053 ESTs of the *Miichthys miiuy*. Out of 123 EST derived microsatellites for which PCR primers were designed, 30 loci were polymorphic in 30 individuals from a single natural population with 2–13 alleles per locus. The observed and expected heterozygosities were from 0.1024 to 0.7917 and from 0.2732 to 0.8845, respectively. Nine loci deviated from the Hardy-Weinberg equilibrium, and linkage disequilibrium was significant between 22 pairs of loci. These polymorphic microsatellite loci will be useful for genetic diversity analysis and molecule-assisted breeding for *M. miiuy*.

## 1. Introduction

Miiuy croaker, *Miichthys miiuy*, is a promising marine fish species for culture in China and is distributed throughout eastern China ([1–3]. Although it is an important commercial fish species, little is known about the genetic information of miiuy croaker. There are no abundant molecular markers such as microsatellites isolated from this species. Lack of enough polymorphic molecular markers has limited development of molecular phylogeny, population structure, and conservation genetics and assisted selective breeding in this species. Thus, screening for polymorphic microsatellite or other molecular markers is necessary for analyzing genetic information in the miiuy croaker. Microsatellites are useful molecular markers to study population structure and genetic evolutionary information [4]. We have published 12 polymorphic microsatellite markers derived from two genomic libraries [5]. Up-to-date, only a few microsatellies markers are available for research in miiuy croaker.

There are many approaches for the development of microsatellite markers such as screening DNA or cDNA libraries for repeat motifs using hybridization and sequencing candidate clones [6], isolation from randomly amplified polymorphic DNA products [7], bioinformatic mining from database [8], *etc*. In general, the development of microsatellite markers has been limited by the labor and time required to construct, enrich, and sequence genomic libraries [9]. However, the development of microsatellite markers from expressed sequence tag (EST) database provides a rich source of valuable functional molecular markers. Herein, 30 polymorphic microsatellite markers were developed by bioinformatic mining EST sequences from *M. miiuy*.

## 2. Materials and Methods

We have constructed a normalized cDNA library from the spleen of the miiuy croaker. A total of 5053 ESTs from the library were sequenced [10]. The EST sequences were screened for mono-, di-, tri-, tetra-, penta-, and hexanucleotide repeats, 491 sequences contained repeat motifs. Primers for these partial loci were designed using PRIMER PREMIER 5.0 software (PREMIER Biosoft International, CA, USA). One hundred and twenty-three primer pairs were designed successfully. Some possessed only few repeats, which held less potential for useful polymorphism.

Genomic DNA was prepared from 30 individuals of miiuy croaker were captured from the Zhoushan fishing ground of the East China Sea. Total genomic DNA was extracted from gills using the TIANamp Genomic DNA Kit (Tiangen) following the manufacturer’s instructions. PCR amplifications were carried out in 25-μL volumes containing 2.5 μL of 10× PCR buffer, 1.5 mM MgCl_2_, 0.2 mM dNTP_S_, 0.2 μM of the forward and reverse primers, and 1.5 units of *Taq* polymerase (Takara). Cycling conditions were 94 °C for 4 min followed by 30 cycles of 94 °C for 40 s, annealing temperature for 45 s (see Table 1), and 72 °C for 40 s, followed by 1 cycle of 72 °C for 5 min and then holding at 4 °C. PCR amplification was performed on an ABI 9700 thermal cycler. Denatured amplified products were separated on 6% denaturing polyacrylamide (19:1 acrylamide:bis-acrylamide) gels using silver staining [6]. A denatured pBR322 DNA/*Msp*I molecular weight marker (Tiangen) was used as a size standard to identify alleles. POPGENE32 [11] and ARLEQUIN 3.11 software [12] were used to calculate the number of alleles, observed (*H*_O_) and expected (*H*_E_) heterozygosity, violation of Hardy-Weinberg equilibrium (HWE) expectations and genotypic linkage disequilibrium. All results for multiple tests were corrected using sequential Bonferroni correction [13].

## 3. Results and Discussion

Details of the newly developed micorastellite loci and variability measures are summarized in Table 1. In total, 30 of 123 loci were successfully amplified and shown to be polymorphic in miiuy croaker. The number of alleles per locus ranging from two to thirteen, and observed and expected heterozygosities ranged from 0.1024 to 0.7917 and from 0.2732 to 0.8845, respectively. The remaining 93 loci were no products or monomorphic in miiuy croaker. Nine loci significantly deviated from Hardy-Weinberg equilibrium in the sampled population after sequential Bonferroni correction (*P* < 0.0017), possibly due to the presence of null alleles, it is thought that these null alleles were caused by genetic instability within this region, the remaining 21 loci conformed to HWE. Further, null alleles were found in twenty-two loci (Table 1) and stuttering were found in nine loci (Mimi-16-A03, Mimi-21-G10, Mimi-28-G08, Mimi-29-C05, Mimi-32-B08, Mimi-36-C02, Mimi-40-E05, Mimi-41-E11, and Mimi-52-H10) detected with MICRO-CHECKER utility after Bonferroni correction [14], but no evidence for allelic dropout were found in any of the loci. In total, 24 pairwises (Mimi-16-E10 and Mimi-5-B04, Mimi-16-E10 and Mimi-13-G10, Mimi-16-E10 and Mimi-21-G10, Mimi-49-C10 and Mimi-21-G10, Mimi-5-B04 and Mimi-21-G10, Mimi-16-A03 and Mimi-21-G10, Mimi-16-H01 and Mimi-21-G10, Mimi-49-C10 and Mimi-32-A10, Mimi-16-H01 and Mimi-32-A10, Mimi-21-G10 and Mimi-32-A10, Mimi-32-A10 and Mimi-34-A09, Mimi-34-A09 and Mimi-35-E08, Mimi-4-C07 and Mimi-36-C02, Mimi-35-E08 and Mimi-40-H12, Mimi-36-C02 and Mimi-40-H12, Mimi-35-E08 and Mimi-41-E11, Mimi-36-C02 and Mimi-41-E11, Mimi-49-C10 and Mimi-54-D06, Mimi-32-A10 and Mimi-54-D06, Mimi-32-B08 and Mimi-54-D06, Mimi-35-E08 and Mimi-57-A05, Mimi-36-C02 and Mimi-57-A05, Mimi-40-H12 and Mimi-57-A05, Mimi-41-E11 and Mimi-57-A05) significant genotypic linkage disequilibrium were found among 285 pairs of the 30 loci after Bonferroni correction (*P* < 0.0017).

## 4. Conclusions

In the present study, 30 polymorphic microsatellite DNA markers were developed by cDNA sequences. These polymorphic microsatellite loci in miiuy croaker will enable studies of the genetic variation, population structure, conservation genetics and molecular assisted selective breeding of the miiuy croaker in the future.

## Figures and Tables

**Table 1:** Characterization of 30 polymorphic expressed sequence tags (EST)-derived microsatellite markers in *M. miiuy*.

Locus	GenBank Accession No.	Repeat Motif	Gene	Primer (5′-3′) [Forward (above) and Reverse (below)]	*T*m (°C)	No. of Alleles	Size range (bp)	No. of Null Alleles	*H*_O_ *H*_E_	*P*-Value
Mimi-4-C07	GW668081	(GAA)_5_	Ras-related protein Rab-35	TGAGGCACAATATGATGG ACCGAGGACTTGGCTACT	52	5	249–288	1	0.1481 0.2732	0.0286
Mimi-5-B04	GW668148	(AGTCAG)_3_	unknown	CTACCGCTGCTCTTCTGG GATGGCTGGTCTACTTCG	49	4	144–162	0	0.4286 0.4662	0.0143
Mimi-5-G02	GW668197	(AGA)_5_	NADH-cytochrome b5 reductase 2	TGTCCGTGCTGTTCTTCC ATGGCTTATGTCCTGTTTCT	49	5	157–169	0	0.2800 0.3502	0.5507
Mimi-8-D03	GW668391	(T)_14_	unknown	TTCAGTCAGGAGATTCAGGGTG CAGCGGTTCAAACGGTCA	48	6	119–128	1	0.4231 0.7360	0.0020
Mimi-13-G10	GW668718	(TTTG)_5_	unknown	GCGACAACGCAGACAGGA CTTGGGCGGATGGTAGGA	52	3	108–116	0	0.5217 0.6309	0.1552
Mimi-16-A03	GW668869	(T)_15_	Cytochrome c	TGGAGAACCCAAAGAAAT CCACAAAGGAGCGTCATA	52	7	282–297	1	0.3793 0.8119	0.0000 *
Mimi-16-E10	GW668916	(TAGCT)_5_	unknown	GTTCTTTCACTGGCATCT GCTGTTTCCACCTGTTTT	50	6	189–224	1	0.4483 0.6062	0.0262
Mimi-16-H01	GW668939	(T)_12_	unknown	CAGTTGTGGGTTTGTTTG TGTGGCGATGTTTCTTGT	52	7	137–150	1	0.5909 0.8478	0.0117
Mimi-21-G10	GW669314	(TTTAT)_3_	phosphatidic acid phosphatase type 2B	GAGCGGGCTTTCCATTCA TTCCCAAATCTGGTGTCTCG	52	2	177–182	1	0.2222 0.3522	0.0636
Mimi-28-G08	GW669768	(A)_14_	unknown	GGGGAAGCACTTTATG TCTTAGCGTGTTCTCGT	52	5	199–203	1	0.1538 0.6380	0.0000 *
Mimi-29-C05	GW669810	(AGG)_5_…(T)_16_	similar to transmembrane protein	AGCCCTCCTCTGCTGTGA CTGTTGCCTCCTGCCTGT	52	5	119–126	1	0.2759 0.5590	0.0311
Mimi-32-A10	GW669955	(A)_14_N_12_(T)_17_	Transmembrane protein 32 precursor	GAACCACCCATCCTTTTA CTTTGCCCCTTCTGTCTA	52	6	226–246	1	0.4348 0.7739	0.0008
Mimi-32-B08	GW669962	(A)_14_…(T)_14_	unknown	CGTCGCACCAAGAATGAG TGAAACCTACCGTCTACAAAT	50	5	236–245	1	0.3846 0.7398	0.0006 *
Mimi-33-G06	GW670085	(CT)_10_N_20_(CA)_9_	unknown	GGTAGGAGACTGGGTGGT CAATGTTTCAGGCAAATGTA	50	5	259–279	1	0.4815 0.6723	0.0581
Mimi-34-A09	GW670103	(A)_13_	unknown	TTTGGGTCACTAAATGGT CGTCTGTAAAGCAGGTAA	50	6	221–242	1	0.5172 0.7992	0.0244
Mimi-35-E08	GW670215	(T)_12_	unknown	ACGCACCCAACAACTCAG ATGCTCATCTCCGCCTTA	50	3	175–182	1	0.1923 0.3288	0.0995
Mimi-36-C02	GW670261	(TTTTC)_3_	ATPase, Ca^++^ transporting, plasma membrane 1a	AATATCCCTGCCCTGCTA TGTTCGCCATTGTCTTGC	50	4	207–227	1	0.1034 0.3575	0.0001 *
Mimi-40-C05	GW670563	(A)_13_	unknown	GTGTAACAAATAACCCTCG TGCTGCTCGTCACAATAA	50	4	131–143	1	0.4800 0.7224	0.0152
Mimi-40-E05	GW670585	(AAT)_5_	Krueppel-like factor 6	AGGGCTCTGATCCATACA TTCCGAAGTGCTCTACAA	50	6	219–243	1	0.1333 0.4418	0.0037
Mimi-40-H12	GW670618	(CCT)5	unknown	TCATCAGCACCAGCCTCT CACATCCTCTTACCTCCTATCT	55	3	233–239	0	0.3704 0.3934	0.0136
Mimi-41-E11	GW670665	(GAA)_5_	unknown	CCTCCTTCACCTCACCTT ACATCTGTCCAGCCGTTT	52	3	238–244	1	0.1379 0.4120	0.0002 *
Mimi-42-E04	GW670734	(ATA)_7_	interleukin-8 receptor CXCR1	CATTCATCACGGCTCCTT TTCCCACTCTTATCTATCCA	48	6	163–181	0	0.7200 0.8196	0.1213
Mimi-42-G06	GW670752	(TCC)_6_	unknown	TTGTTGTCTCGGTGATGG GACTCCTGCTGTTGCTCC	52	6	139–181	0	0.3750 0.4787	0.4739
Mimi-43-H04	GW670839	(TTTC)_6_	unknown	GCTTCCTGTCCCGTTTAT TTTGCTCCCGTGGGTTAT	52	13	141–217	1	0.6552 0.8845	0.6188
Mimi-49-C10	GW671186	(A)_26_	eIF5A	CGGCTTTACTTCAGTGGTT TCTCCTCCTCGGTTGTCG	54	7	180–190	1	0.4583 0.8032	0.0192
Mimi-52-H10	GW671455	(GA)_9_(CTGT)_4_… (T)_14_	unknown	ACGCATTTGTTTACTTTCTC CACCACCATTCAGTTTCT	50	4	188–202	1	0.4074 0.7939	0.0001 *
Mimi-54-A11	GW671541	(CTGGTC)_6_	unknown	AACCAAAGGGACCAAACG GGAGCAGGCAGGTAAACG	52	5	128–152	0	0.6207 0.7042	0.0000 *
Mimi-54-D06	GW671567	(T)_13_…(A)_15_	unknown	TCCTCCCATACAAACTAA GGTGGAAGACCGAAAA	50	3	159–163	0	0.5769 0.6750	0.0000 *
Mimi-56-G05	GW671751	(AGC)_5_	unknown	AGACACCCGACCAGAACC ACAGCCTCCATCCACAAA	54	4	154–160	0	0.7917 0.6764	0.5599
Mimi-57-A05	GW671772	(T)_14_	unknown	CTCCTGCCCTTCGTGATT TCTTTCCCTGCTTGTTGTA	50	6	113–133	1	0.1429 0.4292	0.0011 *

*H*_O_: Observed heterozygosity; *H*_E_: Expected heterozygosity; *T*m: Annealing temperature;

* indicates significant deviation from HWE after Bonferroni correction (*P* < 0.0017).
